# Retraction Note: The majority of conversion total hip arthroplasties can be considered a primary replacement: a matched cohort study

**DOI:** 10.1186/s40001-021-00572-0

**Published:** 2021-09-08

**Authors:** Soufiane Aharram, Mounir Yahyaoui, Jawad Amghar, Abdelkarim Daoudi, Omar Agoumi

**Affiliations:** 1 Department of Trauma and Orthopaedics, University Mohammed Premier Oujda, Mohammed VI Oujda Morocco Hospital Center, Oujda University, BP 4806, 60049 Oujda, Morocco; 2 Faculty of Medicine and Pharmacy of Oujda, Department of Traumatology Orthopeadic Surgery, CHU Mohammed VI, Oujda University, BP 4806, 60049 Oujda, Morocco

## Retraction to: Eur J Med Res (2020) 25:69 10.1186/s40001-020-00467-6

The Editor-in-Chief has retracted this Article due to substantial overlap with an unpublished manuscript by Dr. Georges Vles and colleagues. In addition to this, there is substantial overlap with another article by Aharram et al. which was under consideration at the same time [1].

None of the authors have responded to any correspondence from the editor or publisher about this retraction.
